# A new proposal for secondary surveillance following potentially curative therapy of HCC: alternating MRI and CEUS

**DOI:** 10.1007/s00261-021-03331-1

**Published:** 2021-11-20

**Authors:** Sanjay Bansal, Fangshi Lu, Levi Frehlich, Jason K. Wong, Kelly W. Burak, Stephanie R. Wilson

**Affiliations:** 1 Department of Radiology, University of Calgary, Calgary, AB Canada; 2 Department of Community Health Sciences, University of Calgary, Calgary, AB Canada; 3 Department of Gastroenterology, University of Calgary, Calgary, AB Canada; 4 Department of Diagnostic Imaging, Foothills Medical Centre, 1403 29 St NW, Calgary, AB T2N 2T9 Canada

**Keywords:** Hepatocellular carcinoma (HCC), Contrast-enhanced ultrasound (CEUS), Magnetic resonance imaging (MRI), Hepatobiliary

## Abstract

**Purpose:**

A high recurrence rate following ablative therapy of hepatocellular carcinoma (HCC) necessitates routine follow-up imaging (secondary surveillance) to facilitate early re-treatment. We evaluate our unique secondary surveillance algorithm (with use of alternating MRI and CEUS) by assessment of the relative diagnostic accuracy of MRI and CEUS in detection of residual/recurrent tumor. Potential benefits of alternating surveillance are compared to the use of MRI alone.

**Materials and methods:**

This prospective observational IRB approved study included 231 patients with 354 treated tumors between January 2017 and June 2020. Treated lesions underwent secondary surveillance for a minimum of 7 months and up to 3 years, median follow-up 14 months. Secondary surveillance involved MRI performed at 1 month after treatment, followed by CEUS and MRI at alternate 3-month intervals (i.e., CEUS at month 4, MRI at month 7, etc.), for a total of 2 years. An equivocal finding on one imaging modality triggered expeditious evaluation with the alternate modality. Arterial phase hyperenhancement and washout comprise the classic features of recurrent tumor on both modalities.

**Results:**

A total of 746 MRI and 712 CEUS examinations were performed, and a total of 184 tumor recurrences detected, MRI (*n* = 82) and CEUS (*n* = 102) (*p* = 0.19). There was no difference in the sensitivity (71.0–85.0% and 80.9–92.0%), specificity (97.4–99.2% and 98.5–99.9%), and area under the ROC curve (0.85–0.92 and 0.91–0.96) between MRI and CEUS, respectively. 23 of 82 recurrent tumors identified on MRI were equivocal and confirmed with expedited CEUS. 9 equivocal cases on MRI were disproved by expedited CEUS. On CEUS, 1 of the 102 recurrent tumors was equivocal and confirmed on MRI, and 2 equivocal CEUS cases were disproved by MRI.

**Conclusion:**

MRI and CEUS performed similarly in our secondary surveillance algorithm for HCC in their ability to detect tumor recurrence, and showed no significant difference in their relative diagnostic test accuracy measures. Of greater interest, equivocal results on MRI (typically due to difficulty in distinguishing tumor recurrence from post-treatment change/shunting) were either confirmed or disproven by CEUS in all cases. Secondary surveillance of treated HCC with alternating MRI and CEUS shows equivalent performance of each modality. CEUS resolves equivocal MRI and optimally demonstrates APHE and washout in tumor recurrence.

**Graphic abstract:**

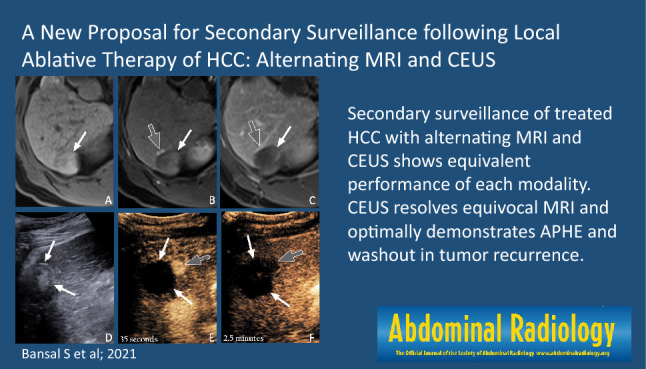

## Introduction

The past decades have been witness to a tremendous improvement in the long-term survival of patients with hepatocellular carcinoma (HCC) [1]. The major reason for this includes the successful implementation of primary surveillance for high risk patients, generally performed with grayscale ultrasound every 6 months [2]. This facilitates early tumor detection, and therefore earlier intervention. Additionally, the emergence of minimally invasive image-guided therapies has facilitated the treatment of non-surgical candidates (nearly 90% of patients), for whom previously a diagnosis of HCC was indicative of impending doom [3]. After decades of painstaking effort at successful management of primary HCC, an emerging challenge relates to tumor recurrence, which occurs in approximately 50% of patients following potentially curative therapy and is prognostically detrimental [4].

The high likelihood of tumor recurrence following initial therapy has prompted major international liver societies, including the American Association for Study of Liver Diseases (AASLD), the European Association for the Study of the Liver (EASL), and the Asian Pacific Association for the Study of the Liver (APASL), to formulate guidelines for post-treatment imaging follow-up [5–7]. These “secondary surveillance” guidelines include recommendations on timing and choice of imaging modality following HCC treatment, with the objectives of early detection and re-treatment of residual/recurrent tumor, which has ultimately been shown to improve long-term survival [4, 8]. Recommendations from the major international liver societies for secondary surveillance include imaging follow-up every 3–6 months with either computed tomography (CT) or magnetic resonance imaging (MRI) [5–7]. Contrast-enhanced ultrasound (CEUS) meanwhile, only recently approved in North America for liver imaging, is suggested as a possible second-line diagnostic tool as its ability to evaluate the entire liver is generally felt to be limited [5–7].

**Fig. 1:**
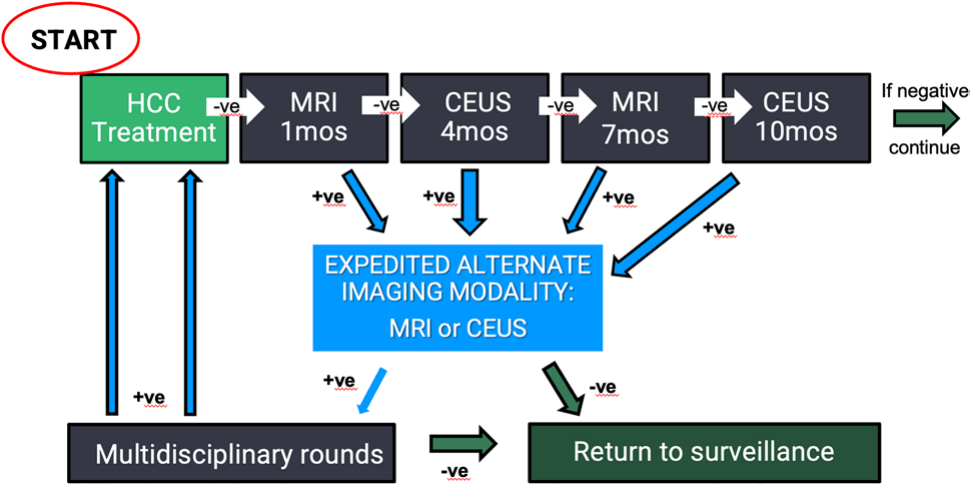
Our local secondary surveillance imaging schedule following HCC therapy Continuous vertical column on the left indicates negative surveillance. Expedited MR/CEUS (middle column) occurs as a result of any positive finding during secondary surveillance. Solid arrows with (-) indicate negative imaging surveillance. Dashed arrows with (+) indicate positive imaging requiring further management

Our diagnostic imaging team in a large tertiary center has more than a decade of experience with CEUS for imaging of HCC, and is integrally involved in weekly multidisciplinary rounds for management of complex patients. With regards to secondary surveillance, initially we used CEUS primarily in conjunction with MRI to resolve indeterminate findings. Over many years, CEUS and more importantly, the powerful combination of MRI and CEUS, were recognized as having an invaluable contribution to the secondary surveillance of HCC. This led to institutional implementation of a unique alternating secondary surveillance algorithm (Fig. 1) in approximately 2016. In this algorithm, we perform MRI one month after HCC treatment, followed by CEUS and MRI performed at alternating three-month intervals.

The current manuscript is the culmination of a multi-year prospective evaluation of our unique alternating secondary surveillance algorithm, in which our objectives are to assess the relative diagnostic accuracy of MRI and CEUS in detection of residual or tumor recurrence, and additionally to identify and describe potential benefits of alternating secondary surveillance compared to secondary surveillance with MRI alone.

## Materials and methods

### Study design

This is a prospective observational IRB approved study, in which written consent was obtained from all enrolled patients. We collected data at our institution from January 2017 to June 2020, on patients who were treated for primary or recurrent HCC with potentially curative treatment including surgical resection or percutaneous ablation (percutaneous ethanol injection, radiofrequency ablation, and microwave ablation), and subsequently underwent alternating secondary surveillance for a minimum of 7 months. This interval of follow-up was chosen to allow ample time for tumor recurrence to occur, and to ensure relative equality in the number of MRI and CEUS exams performed. There were no other specific inclusion criteria. Patients were excluded if they underwent TACE at the time of recruitment, or if they were to have secondary surveillance exclusively with a single modality (i.e., MRI/CT only or CEUS only). Lesions were diagnosed as HCC by meeting criteria for LI-RADS 5 (in the appropriate patient population) or percutaneous biopsy. Patients were not excluded based on prior HCC treatment, nor if a LI-RADS 5 lesion was found to be a non-HCC lesion (i.e., ICC [intrahepatic cholangiocarcinoma] or mixed ICC/HCC) on the pathologic specimen acquired at the time of treatment.

Patients were recruited following multidisciplinary hepatobiliary rounds, where all patients with a new diagnosis of HCC are presented for determination of optimal management. Secondary surveillance was performed as per our local schema (Fig. 1), which entails an MRI performed 1 month after treatment, followed by CEUS and MRI performed at alternating 3-month intervals (i.e., CEUS at month 4, MRI at month 7, etc.), for a total of 2 years. Patients who were recurrence free for two years after treatment were discharged to the community, where they were evaluated with grayscale ultrasound only, at an increased follow-up interval of 6 months. Patients with new or recurrent tumors within two years of treatment, were typically re-treated and included in the data as a new lesion. In accordance with our local practice, an equivocal finding on one imaging modality during secondary surveillance triggered expeditious evaluation with the alternating imaging modality (Fig. 1). All cases interpreted as “equivocal” or “positive for tumor recurrence” were discussed at multidisciplinary hepatobiliary rounds to determine management. In order to evaluate our real-world clinical practice, image interpretation was based on the report of the dictating radiologist (all of whom were abdominal imaging specialists with experience ranging from 2 to 15+ years) unless an alternate decision was rendered at multidisciplinary hepatobiliary rounds. Access to clinical, biochemical, and prior imaging information was available and its use was at the discretion of the multidisciplinary team. The performance of our study did not influence image interpretation or decisions regarding patient management.

### Imaging techniques

The CEUS technique for this study was highly standardized, performed at a sole institution, and interpreted by a group of 6 abdominal radiologists. Of the 6 radiologists that interpreted CEUS, 2 solely interpreted CEUS and did the majority of scans, while the remaining 4 interpreted both CEUS and MRI. The MRI technique was also standardized, but by comparison was performed at four University affiliated facilities, and the group of interpreting radiologists was larger (approximately 15–20 radiologists, all of whom were Abdominal Imaging specialists).

#### CEUS technique

The majority of exams were performed with either an RS80 EVO ultrasound machine (Samsung, Seoul, South Korea) or an ACUSON Sequoia (Siemens, Munich, Germany). A radiologist was typically not present in the room with the technologist for performance of the examination, but images were checked with the radiologist for all cases prior to discharging the patient from the department. However, the radiologist’s involvement increased for more complex cases. For performance of contrast-enhanced ultrasound, the machine must be equipped with the following:

- Contrast-specific software to enable:
  - Production of low mechanical index sound waves (to minimize destruction of the microbubble contrast agent)
  - Real-time subtraction technique (to create a microbubble only image)
- Dual-screen mode (with dual screen calipers) for anatomic correlation between the grayscale image and the subtracted microbubble only image
- Clearly visible on-screen timer

We typically use perflutren microspheres (Definity; Lantheus Medical Imaging, Billerica, MA), a purely intravascular microbubble contrast agent, which functionally is indistinguishable from sulfur hexafluoride (Lumason; Bracco Diagnostics Inc., Monroe Township, New Jersey).

Prior to initiating the exam, the patient’s treatment history must be obtained. Vital information includes treatment type, treatment date, treatment site location, pretreatment imaging features of lesion, and any prior images of the treatment site.

Next, the patient’s liver is thoroughly evaluated with grayscale ultrasound. This very important step serves multiple purposes. Firstly, all known treatment sites are identified, measured, and correlated with known treatment history. It is essential to assess the treatment site for any juxtaposed areas of nodularity on grayscale. Secondly, any new or growing nodules elsewhere in the liver are identified. Lastly, the grayscale ultrasound is used to plan the contrast-enhanced ultrasound. Planning consists of identifying the best possible acoustic window, best patient position, and practicing the required breathing technique with the patient to facilitate optimal evaluation of the treatment site. We prefer assessment in the long-axis to reduce in-plane and out-of-plane respiratory motion, but this is not always possible. During performance of the contrast-enhanced scan the transducer is maintained over the central portion of the treatment site during the arterial phase, with careful cranial and caudal sweeps to assess the entirety of the treatment site.

The set-up for CEUS involves obtaining intravenous access with a 20–24 gauge needle, onto which a three-way stopcock is attached. A 1.2 mL vial of perflutren is activated in a vial mixer (Vialmix; Lantheus Medical Imaging, Billerica, MA), which increases the content volume to 1.8 mL. Perflutren is then drawn into a 1.0 mL syringe and attached to the parallel/straight port of the three-way stopcock. A 10 mL saline flush is attached to the perpendicular/side port. Next, 0.2 mL of perflutren is injected by hand, immediately followed by a 10 mL saline flush (which ensures optimal delivery of the contrast bolus). Occasionally 0.3 mL of perflutren is used for severely cirrhotic livers that enhance poorly.

Timer initiation is simultaneous to initiation of the saline flush. The treatment site is monitored continually in anticipation of the first microbubble, at which time the cine loop recording is initiated. Ongoing continuous monitoring is performed from initiation of the *timer* (not of the cine loop recording) to just beyond the peak of arterial phase enhancement. Subsequently images are taken at 30–60 s intervals, and including at 1 min, to assess for washout up to 5–6 min, with particular attention paid to any suspicious foci of APHE to identify corresponding washout.

Repeat contrast injections are performed, as needed (up to a dose of 20 uL/kg), to re-evaluate any suspicious findings or if the initial assessment is unsatisfactory. Note that a maximal dose of 20uL/kg would permit up to 1.4 mL of total contrast agent (7 injections of 0.2 mL) in a 70 kg patient. Additionally, a technique we refer to as an “on top injection” is particularly useful if a discrete region of washout is observed. In such a circumstance, an additional 0.2 mL of perflutren is injected (followed by 10 mL saline flush) during the portal venous or late phase on top of the identified washout. This valuable technique facilitates careful observation for corresponding APHE in a known region of washout, which may not have been conspicuous on the initial injection.

#### MRI technique

MRI technique varied slightly between institutions and scanners, but all protocols met the requirements detailed in the CT/MRI LI-RADS v2018 manual. All scans were performed on either a 1.5T or 3T magnet. Universally acquired sequences included unenhanced T1-weighted in-phase and out-of-phase images, unenhanced T2-weighted images with and without fat suppression, and fat suppressed multiphase 3D T1-weighted images (pre-contrast, late arterial phase, portal venous phase, and delayed phase) using the macrocylic extracellular contrast agent gadobutrol (Gadavist; Bayer Healthcare Pharmaceuticals, Leverkusen, Germany), and post-processed subtraction images of the fat suppressed multiphase T1-weighted images.

### Image interpretation: MRI and CEUS

**Fig. 2:**
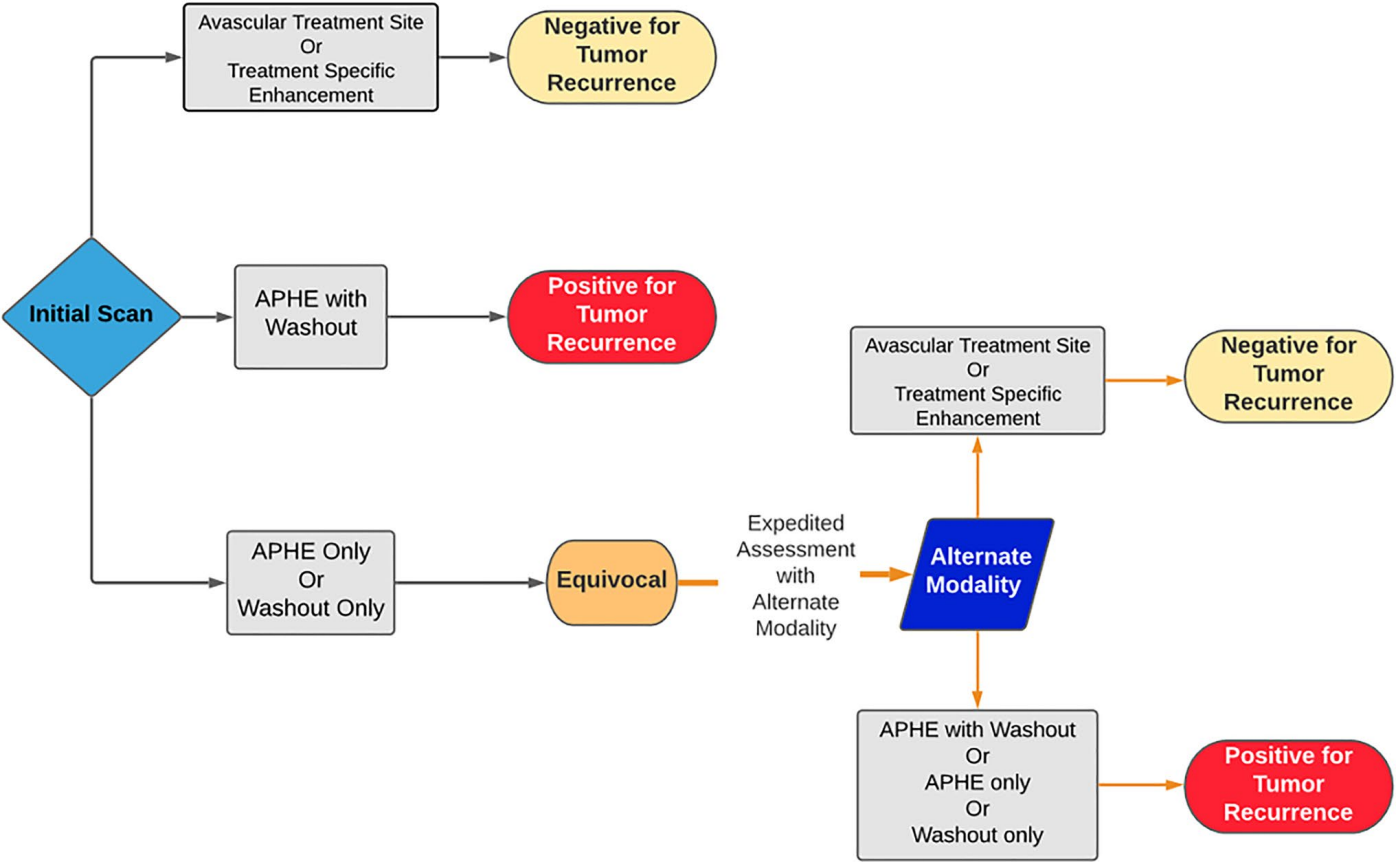
Flowchart summarizing interpretation of imaging findings. An avascular treatment site, or the presence of treatment specific enhancement, was defined as negative for tumor recurrence on both modalities (equivalent to LR-TR Non-Viable). The presence of a nodular region of arterial phase hyperenhancement (APHE) that demonstrates washout on portal venous or delayed phase images was defined as positive for tumor recurrence on both modalities (equivalent to LR-TR Viable). An isolated finding of APHE only or washout only, on either modality, was defined as equivocal for tumor recurrence (equivalent to LR-TR Equivocal). Equivocal findings were further evaluated with the alternative modality. An avascular treatment site or treatment specific enhancement on the alternate modality was then defined as negative for tumor recurrence. APHE with washout, APHE only, or washout only, seen on the alternate modality (and corresponding to the equivocal finding on the first modality) was then defined as positive for tumor recurrence

The algorithm for interpretation of imaging findings is outlined in Fig. 2 and detailed in the following text. These definitions were designated based on our clinical experience, as the onset of our study (2017) predated inclusion of a “Treatment Response” section in the CT/MRI LI-RADS Manual, which was first included in the 2018 version [10].

#### Positive for recurrence

MRI scans were categorized as positive tumor recurrence in the presence of APHE with washout appearance (in portal venous or late phase), or enhancement similar to the initial lesion prior to treatment. We defined recurrence on CEUS in the same way (i.e. APHE with washout, or enhancement similar to pretreatment). This is a deviation from the definition in the “Treatment Response” section of the LI-RADS CT/MRI manual (v2018), which suggests APHE, or washout appearance, or enhancement similar to pre-treatment is sufficient to diagnose tumor recurrence (i.e. “LR-TR Viable”) [10].

#### Equivocal for recurrence

Cases in which APHE or washout occurred as a solitary finding on either modality, or enhancement was atypical for post-treatment appearance, were categorized as equivocal. Following expeditious assessment with the alternate modality the equivocal result was either confirmed or disproven (Fig. 2). The following findings on the alternate modality were considered confirmatory for tumor recurrence: APHE with washout, or APHE only, or washout only. An avascular treatment site or treatment-specific enhancement were considered negative for tumor recurrence. The LI-RADS manual defines an equivocal result (LR-TR Equivocal) as “enhancement atypical for treatment-specific expected enhancement pattern and not meeting criteria for probably or definitely viable” [10].

#### Negative for recurrence

A scan was categorized as negative for tumor recurrence in the setting of an avascular treatment site or treatment-specific enhancement pattern. This is identical to the definition used by LI-RADS (LR-TR “Non-Viable”) [10].

#### Geographic categorization of recurrent tumor

**Fig. 3:**
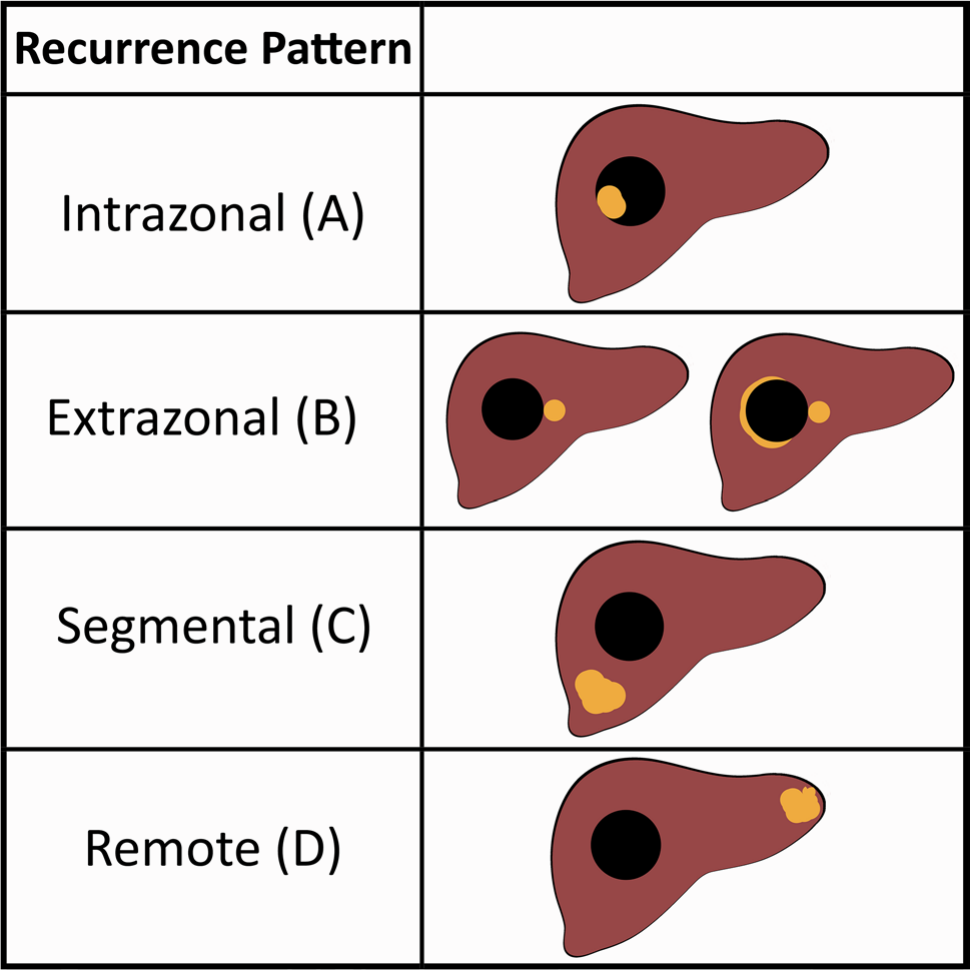
Schematic representation shows geographic HCC recurrence patterns (orange). Reproduced from reference 9 with permission from The Radiologic Society of North America (RSNA). Intrazonal tumor occurs within a treatment site. Extrazonal tumor describes recurrent tumor that is juxtaposed to, but outside of, the treatment site. Segmental tumor occurs within the same segment as a previously treated lesion, but with normal intervening liver tissue, and may represent either a satellite nodule or de novo HCC. Remote recurrence is a new nodule, in a segment separate from any treatment site. Intrazonal and extrazonal tumors are considered true recurrence (i.e. LR-TR Viable), while segmental and remote tumors are considered new tumors (i.e. LR-4 or LR-5 lesion)

Recurrent tumors were categorized based on their location relative to the treatment site as intrazonal or extrazonal (Fig. 3) [9]. Newly identified nodules were categorized as segmental (within the same segment as a treatment site), or as remote (elsewhere in the liver). Geographically describing tumor recurrence has over time become integral to interactions with our Interventional Radiology colleagues, as it contributes to decision-making at multidisciplinary rounds and treatment planning.

As compared to standard liver ultrasound, where nodules are measured on grayscale images, post-treatment scans are better viewed in conjunction with the contrast-enhanced image. This is because the exact margins of both the treatment site and recurrent tumor are often not clearly delineated on the grayscale images alone. The size of recurrent tumors was measured based on the longest dimension, in accordance with the LI-RADS CT/MRI Manual [10].

### Statistical analysis

Patients with incomplete treatment were not included in the analysis, until they were re-treated and complete treatment was achieved. Additionally, the first follow up was not included in the test comparisons, as it was constrained to MRI only. Subsequent alternating total number of MRI and CEUS examinations were analyzed using classical test diagnostics.

Point and 95% confidence interval (CI) estimates were calculated for sensitivity, specificity, and the receiver operator curve (ROC). A scan was denoted as true positive (TP) if it met requirements for “positive for recurrence” (defined in previous section), or if it was “equivocal for recurrence” and subsequently confirmed as “positive for recurrence” on the expedited scan. If a scan was “equivocal for recurrence” and disproven (i.e. “negative for recurrence”) on the expedited scan, it was denoted as false positive (FP). When a scan was “positive for recurrence”, the preceding scan was re-examined. If, in retrospect, tumor recurrence was clearly visible on the preceding scan the scan was denoted as false negative (FN). If tumor could not be clearly identified on the preceding scan, it was denoted as true negative (TN). True negative (TN) denotation was also applied to scans that were “negative for recurrence”, and remained “negative for recurrence” on at least 2 follow-up scans. All statistical analyses were undertaken using Stata ® version 16.1 (StataCorp, TX, USA) with an alpha of 0.05.

## Results

Table 1 summarizes the demographic and treatment characteristics of the patient cohort. A total of 252 patients were initially recruited, however 21 patients were lost to follow-up. The remaining 231 patients, in whom there were a total of 240 tumors at the time of recruitment, were all included in data analysis. During secondary surveillance an additional 114 lesions were detected and added to the patient cohort, resulting in a total of 354 treated lesions included in data analysis. Follow-up duration for treated lesions ranged between 7 months and 3 years, with a median follow-up of 14 months. Of the 231 included patients, 66 were female and 165 were male. Mean patient age was 67.8 years. Cirrhosis was present in 207 patients, and 73 patients had previously undergone treatment for HCC. T Of the 354 treated lesions 338 were diagnosed as HCC by meeting criteria for LI-RADS 5, while the remaining were diagnosed by percutaneous biopsy. Of the 338 LI-RADS 5 lesions, tissue sampling was obtained at the time of treatment in 113 lesions yielding the following results: 109 HCC, 2 mixed HCC/ICC, and 2 ICC. Treated lesions were followed for a minimum of 7 months and up to 3 years, with a median follow-up of 14 months. Most lesions were treated with percutaneous ablation (95%), and the remainder were treated with surgical resection. Excluding the 1st follow up a total of 619 MRI and 699 CEUS examinations were performed.

**Table 1:** Demographic and treatment characteristics

	Number of patients (%)
Mean age	67.8 years
Gender	
Male	165 (71%)
Female	66 (29%)
Cirrhosis	207 (90%)
Etiology of chronic liver disease	
Ethanol	64 (28%)
Hepatitis B virus	48 (21%)
Hepatitis C virus	70 (30%)
NAFLD	34 (15%)
Other	15 (6%)
History of treated HCC	73 (32%)
Total number of treated lesions	354
Treatment type	
Percutaneous ethanol injection	51 (14%)
Radiofrequency ablation	138 (39%)
Microwave ablation	146 (41%)
Surgical resection	19 (5%)

A similar number of tumor recurrences were detected on MRI (*n* = 82) and CEUS (*n* = 102). There was no difference in the sensitivity (79.3% [69.6–87.1%] and 86.8% [80.3–91.7%] for MRI and CEUS, respectively), specificity (98.7% [97.3–99.5%] and 99.5% [98.4–99.9%]), and area under the ROC curve/AUC (0.89 [0.85–0.93] and 0.93 [0.90–0.96]) between MRI and CEUS (Table 2). In a sensitivity analysis, inclusion of the first follow up did not change our results.

**Table 2:** Results

	MRI	CEUS	Total
# of exams performed	617	699	1316
Recurrent/new tumors	82	102	184
Recurrent tumors			
Intrazonal	4	27	31
Extrazonal	38	28	62
New tumors			
Segmental	17	12	28
Remote	29	35	63
Test accuracy statistics			
Sens (95% CI)	79.3 (69.6–87.1)	86.8 (80.3–91.7)	
Spec (95% CI)	98.7 (97.3–99.5)	99.5 (98.4–99.9)	
ROC area (95% CI)	0.89 (0.85–0.93)	0.93 (0.90–0.96)	
Test results			
True positive	73	131	
True negative	520	545	
False positive	7	3	
False negative	19	20	

Of the 82 recurrent tumors identified on MRI, 23 were interpreted as an equivocal result and required confirmation with expedited CEUS (Table 3). Most commonly (20/23), MRI showed APHE only and the expedited CEUS showed APHE with washout. The other 3 cases showed APHE only on both modalities. There were 9 cases in which an equivocal MRI (8 cases of APHE only and 1 case of washout only) was disproved by expedited CEUS (avascular treatment site).

**Table 3:** Equivocal results

	Confirmed (TP)	Disproven (FP)	Total
Total	24	11	35
MRI	23	9	32
CEUS	1	2	3
Confirmed cases (true positive results)			
Modality	Number of result	Initial scan finding	Expedited scan finding
MRI	20/23	APHE only	APHE with washout
MRI	3/23	APHE only	APHE only
CEUS	1 / 1	Washout only	APHE with washout
Disproven cases (false positive results)			
Modality	Number of result	Initial scan finding	Expedited scan finding
MRI	8 / 9	APHE only	Avascular
MRI	1 / 9	Washout only	Avascular
CEUS	2 / 2	APHE only	Avascular

On CEUS, 1 of the 102 recurrent tumors was interpreted as equivocal (washout only on CEUS, and APHE with washout on expedited MRI). There were 2 cases in which an equivocal CEUS (APHE only) was disproved by expedited MRI (avascular treatment site).

Intrazonal tumor recurrence was demonstrated more frequently on CEUS (*n* = 27) than on MRI (*n* = 4). All other geographic types of tumor recurrence (i.e. extrazonal, segmental, and remote) were demonstrated in similar proportions on both modalities (Table 2).

## Discussion

**Fig. 4:**
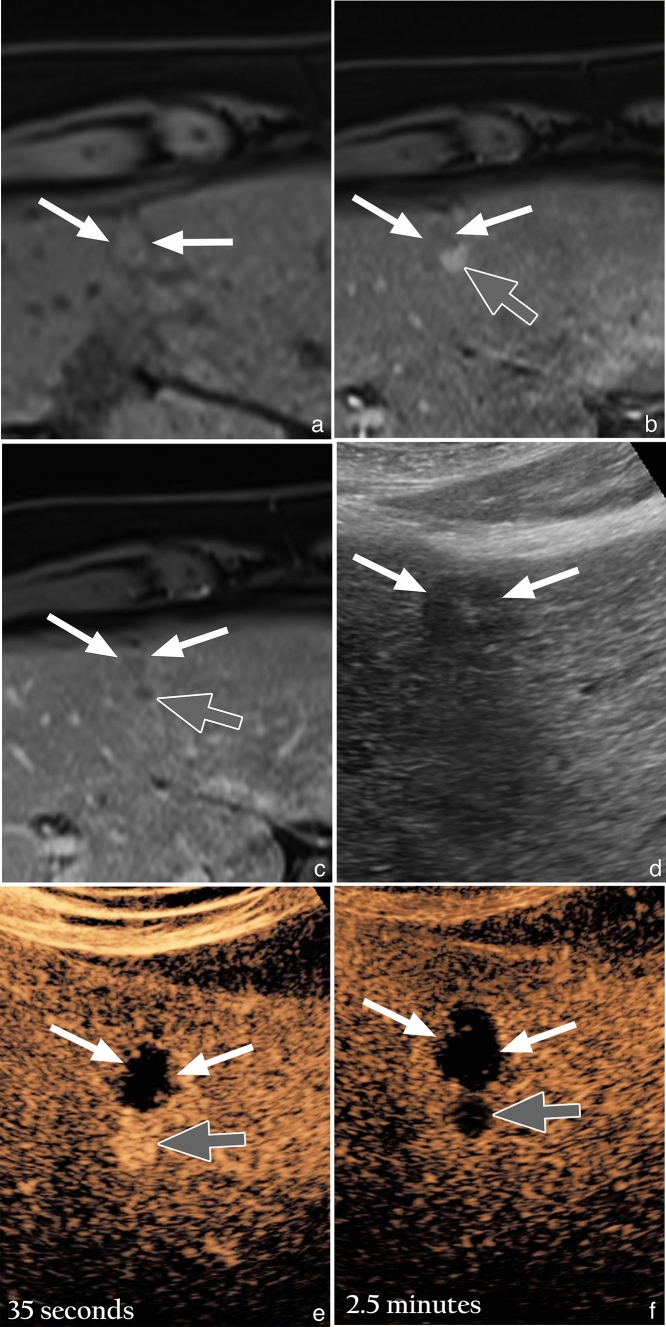
Concordant extrazonal recurrence on MRI and CEUS after MWA in a 54-year-old man with HCV cirrhosis. **a**) Unenhanced T1 weighted fat suppressed (T1FS) image shows an isointense treatment site (white arrows). **b**) Arterial phase T1FS subtracted image shows a nodular area of hyperenhancement (gray arrow), inseparable from the posterior margin of the treatment site (white arrows). **c**) Delayed phase T1FS subtracted image shows washout (gray arrow), corresponding to the nodular area of hyperenhancement seen on the arterial phased image. **d**) Gray-scale US image, in the same patient, shows a hypoechoic treatment site (white arrows). **e**) CEUS image obtained 35 seconds after microbubble contrast injection shows a nodular area of hyperenhancement (gray arrow) inseparable from the avascular treatment site (white arrows), equivalent to the arterial phased MR image. **f**) CEUS image obtained at 2.5 minutes shows definite washout (gray arrow) of the arterially hyperenhancing nodular focus

The results suggest that MRI and CEUS perform similarly in secondary surveillance of HCC as indicated by the similar number of tumor recurrences detected on each modality (82 on MRI and 102 on CEUS) and the overlapping confidence intervals for the calculated sensitivity, specificity, and AUC values (Table 2). These results are in accord with a European study by Catalano et al., which also demonstrated equivalence between CEUS and CT/MRI in alternating modality secondary surveillance, as well as a meta-analysis by Shi et al. that showed equivalence between CEUS and CT/MRI in short-term follow up (< 1 month) [11, 12]. A case of extrazonal tumor recurrence, with concordance on MRI and CEUS, is shown in Fig. 4 for readers who may be unfamiliar with the comparative appearance of tumor recurrence on both modalities.

Of equal, or perhaps greater importance, are the implications gleaned from the equivocal results data (Table 3), which indicate that:

- Equivocal results occurred far more often on MRI as compared to CEUS (32 vs 3)
- The most common equivocal result on MRI was APHE only (28 total cases)
- Isolated APHE on MRI was accurately resolved by CEUS (20 cases showing APHE with washout, 3 cases showing corroborating APHE, and 8 cases showing an avascular treatment site, confirmed on follow-up imaging.

**Fig. 5:**
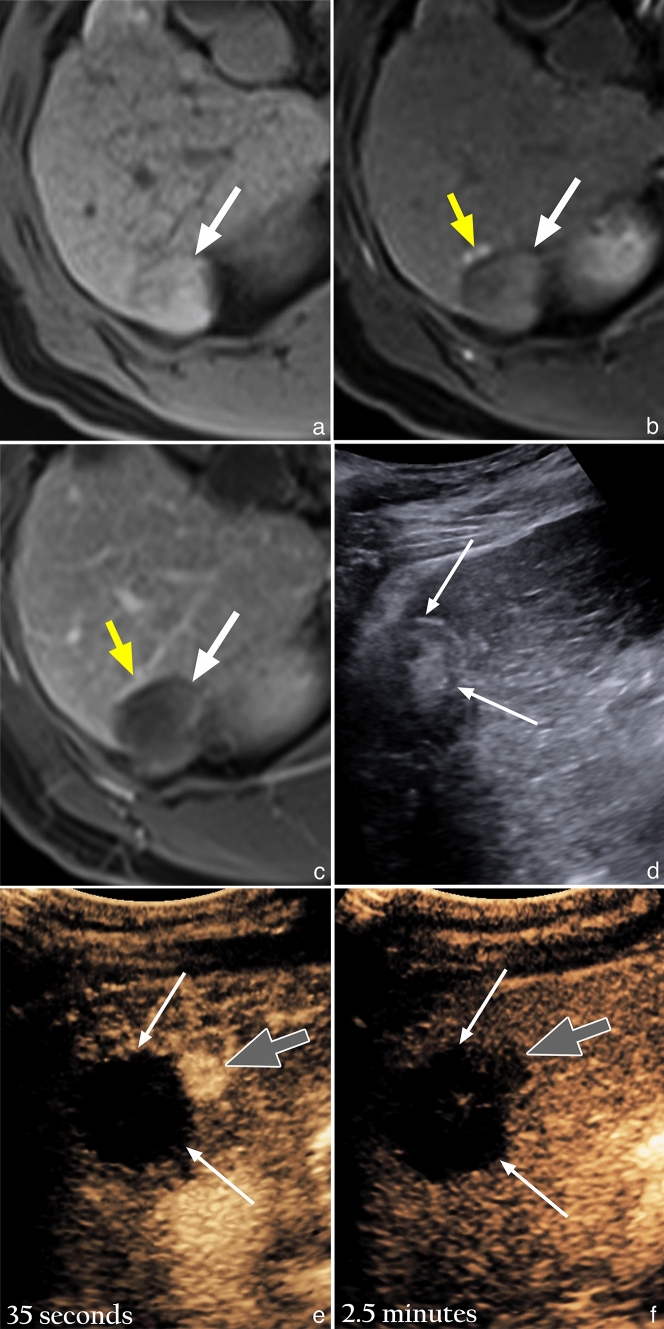
Equivocal extrazonal recurrence on MRI, confirmed on expedited CEUS, after RFA in a 66-year-old woman with ethanol induced cirrhosis. **a**) Unenhanced T1 weighted fat suppressed (T1FS) image shows a mildly hyperintense treatment site (white arrow). **b**) Arterial phase T1FS subtracted image shows a subtle nodular focus of hyperenhancement (gray arrow), along the anterior margin of the treatment site (white arrow). **c**) Delayed phase T1FS subtracted image shows mild persistent enhancement (gray arrow) of the subtle arterially enhancing focus, with no convincing evidence of washout. **d**) Gray-scale US image, obtained 1 week after the MRI, shows a heterogenous treatment site (white arrows). **e**) CEUS image obtained 35 seconds after microbubble contrast injection shows a highly conspicuous nodular area of hyperenhancement (gray arrow) inseparable from the avascular treatment site (white arrows), corresponding to the subtle focus of arterial hyperenhancement seen on the MRI. **f**) CEUS image obtained at 2.5 minutes shows washout (gray arrow) of the arterially hyperenhancing nodular focus

Recent studies from North America as well as Asia, aimed at validation of the LI-RADS treatment response (LR-TR) algorithm on MRI, have shown that the LR-TR Equivocal category is assigned in 11–27% of cases [13–15]. Similarly, in our study 23/82 (28%) of recurrent tumors identified on MRI were initially interpreted in equivocal. The challenge in evaluation of a treatment site on MRI lies in distinguishing tumor recurrence from post-treatment changes/shunting, as both are characterized by APHE and can be quite similar in their appearance [16]. Our data suggest that CEUS is highly effective in its ability to resolve whether APHE on MRI represents tumor recurrence or post-treatment change/shunting. An example case of this is shown in Fig. 5. In our study, an equivocal finding of APHE only on MRI most commonly showed either APHE with washout on CEUS to confirm tumor recurrence (20/32), or an avascular treatment site to disprove tumor recurrence (8/32). This may be explained by the nature of the purely intravascular microbubble contrast agent, which does not undergo extravasation into the interstitium in the presence of a shunt or increased endothelial permeability (as compared to CT and MRI contrast agents which have an interstitial/extracellular phase). Additionally, CEUS has been shown to have a greater sensitivity for detection of small areas of APHE and washout, relative to MRI [17–19]. Other factors that may account for our results include greater spatial and temporal resolution of ultrasound (real time evaluation eliminates issues related to timing and motion artifact), the ability to perform multiple injections (facilitating on the spot re-assessment of any questionable findings), and the CEUS subtraction software. The CEUS subtraction software completely nullifies the pre-contrast image, making any areas of APHE highly conspicuous, as compared to the MRI subtraction technique which only partially nullifies pre-contrast signal and is also prone to misregistration artifact. If maximal specificity is desired, the requirement of APHE and washout for confident diagnosis of tumor recurrence may be considered for the “Treatment Response” section in the next iteration of CEUS LI-RADS, as this combination is present in the majority of cases, and more reliably predicts the presence of viable tumor as compared to APHE or washout alone. However, the trade-offs between sensitivity and specificity must be carefully considered, and dedicated studies correlating imaging criteria with histopathology are needed to validate this statement.

**Fig. 6:**
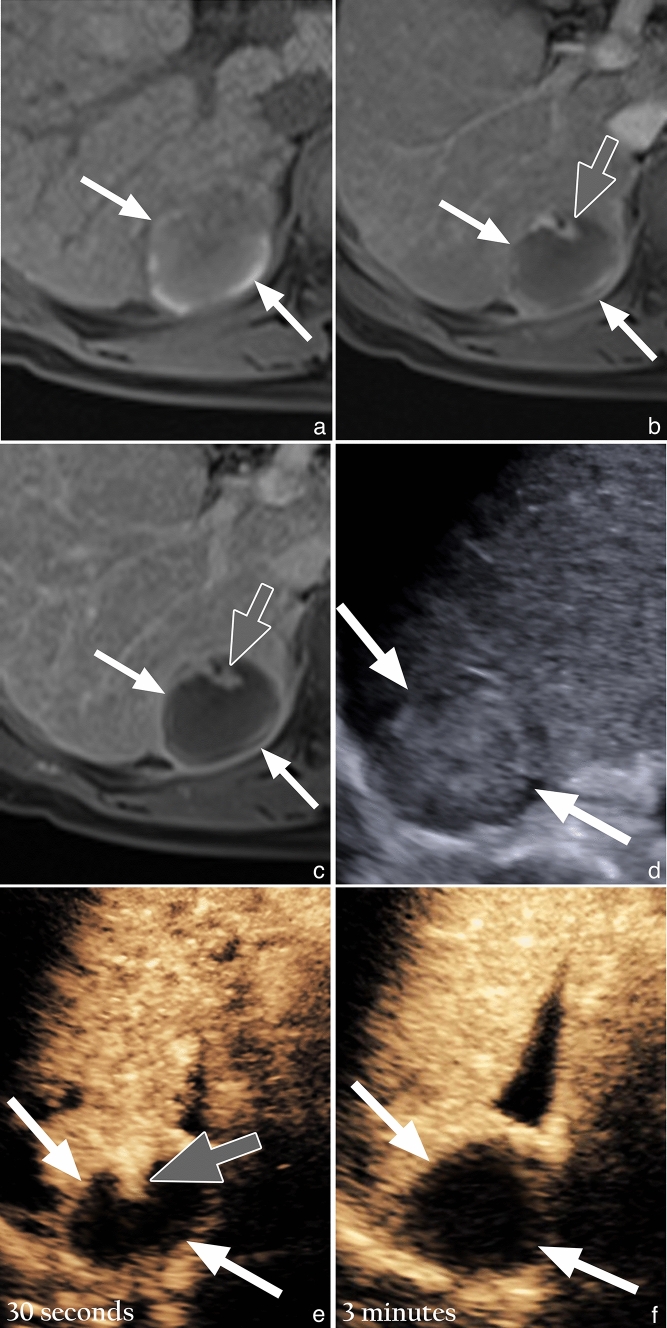
Resolution of the discordant appearance of intrazonal recurrence on MRI and CEUS, after RFA in a 70-year-old man with HBV cirrhosis. **a**) Unenhanced T1 weighted fat suppressed (T1FS) image shows a mildly hypointense treatment site (white arrows) with curvilinear arrows of hyperintensity at the periphery of the treatment site. **b**) Arterial phase T1FS subtracted image shows a mildly hyperenhancing nodular focus (gray arrow), within the anterior aspect of the treatment site (white arrows). **c**) Delayed phase T1FS subtracted image shows persistent enhancement (gray arrow) of the mildly arterially hyperenhancing focus, with no convincing evidence of washout. **d**) Gray-scale US image shows a mildly hyperechoic treatment site (white arrows). **e**) CEUS image obtained 30 seconds after microbubble contrast injection shows a nodular area of hyperenhancement (gray arrow) in the anterior aspect of an otherwise avascular treatment site (white arrows), corresponding to the mildly arterially hyperenhancing focus seen on the MRI. **f**) CEUS image obtained at 3 minutes shows washout of the arterially hyperenhancing nodular focus, in keeping with washout, leaving a completely avascular treatment site (white arrows)

Another interesting point of discussion relates to intrazonal tumors, which were demonstrated more frequently on CEUS (*n* = 27) than MRI (*n* = 4). One reason for this disparity may be due to the heterogeneous appearance of a treatment site on unenhanced T1-weighted MR images, which often contains areas of high T1 signal intensity that are not always completely nullified on subtraction images. In stark comparison, a normal treatment site on CEUS has a completely anechoic appearance due to the subtraction software. Hence, intrazonal recurrence on CEUS tends to be more conspicuous. Furthermore, in our personal experience retrospectively looking at MRI scans performed prior to CEUS scans that show intrazonal tumor recurrence, we observed that intrazonal tumor recurrence on MRI tends to show a persistent enhancement appearance (rather than typical APHE with washout, which is seen on CEUS). An example of this is shown in Fig. 6. A possible explanation for this may be due to extravasation of MRI contrast agents into the interstitium of a treatment site, which is composed largely of necrotic tissue. In contradistinction, as previously mentioned, CEUS contrast agents are entirely intravascular and do not extravasate into the interstitium. This suggests that detection of intrazonal type tumor recurrence may be limited on MRI.

A limitation of this study was the absence of histopathologic confirmation of the imaging findings. Histopathologic confirmation is extremely difficult in non-explanted livers, as recurrent tumors are often small in size and challenging to accurately sample at the time of repeat ablation. Another limitation was that MRI was performed at multiple institutions, whereas CEUS was performed at a single institution, which may have increased heterogeneity in MRI scan quality. However, such is the reality in most practices, as CEUS is a relatively novel technique in North America and has not yet attained the ubiquity of MRI. Furthermore, the potential impact of this limitation may have been at least partially diminished by the fact that all equivocal and positive cases (i.e. both MRI and CEUS) were reviewed at multidisciplinary rounds. Confirmation of tumor with expedited CEUS to clarify an equivocal MRI finding is preferable to ablating a negative tumor, but it is important to consider that from a practical clinical perspective not all equivocal findings require immediate action, and that a 3-month follow-up interval may be of further benefit in determining clinical significance.

## Conclusion

Evaluation of our unique secondary surveillance algorithm of treated HCC, with alternating MRI and CEUS, shows equivalent performance of each modality in their ability to detect tumor recurrence. Of greater interest, we found that equivocal results on MRI (typically due to difficulties in distinguishing tumor recurrence from post-treatment change/shunting) were either confirmed or disproven by CEUS in all cases. Based on our findings, the CEUS LI-RADS committee may consider requiring the presence of APHE and washout for confident diagnosis of tumor recurrence if maximal specificity is desired, as this combination is present in most cases, and more reliably predicts the presence of viable tumor as compared to APHE or washout alone. However, dedicated studies correlating imaging criteria with histopathology are needed to validate this statement.

## Data Availability

Available upon request.
