# Correction to: A new proposal for secondary surveillance following potentially curative therapy of HCC: alternating MRI and CEUS

**DOI:** 10.1007/s00261-021-03385-1

**Published:** 2022-01-08

**Authors:** Sanjay Bansal, Fangshi Lu, Levi Frehlich, Jason K. Wong, Kelly W. Burak, Stephanie R. Wilson

**Affiliations:** 1 Department of Radiology, University of Calgary, Calgary, AB Canada; 2 Department of Community Health Sciences, University of Calgary, Calgary, AB Canada; 3 Department of Gastroenterology, University of Calgary, Calgary, AB Canada; 4 Department of Diagnostic Imaging, Foothills Medical Centre, 1403 29 St NW, Calgary, AB T2N 2T9 Canada

## Correction to: Abdominal Radiol (2021) 10.1007/s00261-021-03331-1

The Article “A new proposal for secondary surveillance following potentially curative therapy of HCC: alternating MRI and CEUS”, written by Sanjay Bansal, Fangshi Lu, Levi Frehlich, Jason K. Wong, Kelly W. Burak, Stephanie R. Wilson, was originally published electronically on the publisher's internet portal on 20 November 2021 without open access. With the author(s)’ decision to opt for Open Choice the copyright of the article changed on 6 December 2021 to © The Author(s) 2021 and the article is forthwith distributed under a Creative Commons Attribution 4.0 International License, which permits use, sharing, adaptation, distribution and reproduction in any medium or format, as long as you give appropriate credit to the original author(s) and the source, provide a link to the Creative Commons licence, and indicate if changes were made. The images or other third party material in this article are included in the article's Creative Commons licence, unless indicated otherwise in a credit line to the material. If material is not included in the article's Creative Commons licence and your intended use is not permitted by statutory regulation or exceeds the permitted use, you will need to obtain permission directly from the copyright holder. To view a copy of this licence, visit http://creativecommons.org/licenses/by/4.0. The original article has been corrected.

